# Molecular characterization and antimicrobial susceptibility for 62 isolates of *Bordetella pertussis* from children

**DOI:** 10.3389/fmicb.2024.1498638

**Published:** 2024-12-11

**Authors:** Baohua He, Zhaoyi Jia, Fei Zheng, Wenchao Zhang, Suxia Duan, Leyu Wang, Haixia Zhang, Hongbin Zhang, Ruoxuan Wang, Yuan Gao, Yinqi Sun

**Affiliations:** ^1^Hebei Key Laboratory of Pathogens and Epidemiology of Infectious Diseases, HeBei Provincial Center for Disease Control and Prevention, Shijiazhuang, China; ^2^School of Public Health, North China University of Science and Technology, Tangshan, China; ^3^Hebei Children's Hospital, Shijiazhuang, China; ^4^National Key Laboratory of Intelligent Tracking and Forecasting for Infectious Diseases, National Institute for Communicable Disease Control and Prevention, Chinese Center for Disease Control and Prevention, Beijing, China

**Keywords:** *Bordetella pertussis*, erythromycin resistance, China, children, *ptxP1*

## Abstract

**Background:**

Pertussis is a highly contagious respiratory disease caused by *Bordetella pertussis* (BP). Despite global control of pertussis cases through the Expanded Programme on Immunization (EPI), there has been a significant increase in the incidence of pertussis in recent years, characterized by a “resurgence” in developed countries with high immunization rates as well as a comparable reemergence in certain areas of China. We aim to explore the genotypes and antimicrobial susceptibility of circulating BP from children in Hebei.

**Study design:**

Children diagnosed with BP infection from 2019 to 2020 in Hebei, China were enrolled. We performed antimicrobial susceptibility testing (AST), whole-genome sequencing (WGS) analysis, single nucleotide polymorphism (SNP) detection, mltilocus sequence typing (MLST), multilocus antigen sequence typing (MAST), multilevel genome typing (MGT). A total of 313 international BP genomes were selected for comparison to examine the genomic diversity and evolutionary traits of Chinese strains within a global framework.

**Results:**

Sixty-two individuals were identified with BP infection via culture, yielding a positive rate of 15.62% (62/397) for BP. Two phylogenetic groups were identified, each carrying a dominating genotype. The two vaccine strains, CS and Tohama I, exhibited a distant relationship to these two groups. This study identified 56 erythromycin-resistant isolates, 55 azithromycin-resistant isolates, 58 sulfamethoxazole-sensitive isolates, and 53 cefotaxime-sensitive isolates. All BP isolates were sensitive to levofloxacin, amoxicillin, ceftriaxone, and meropenem. Meanwhile, all erythromycin-resistant strains, which belonged to lineage I and MGT2 sequence type 7 (ST7), shared the *ptxP1* gene and contained the 23S rRNA A2047G mutation. The major MAST was *prn1*/*ptxP1*/*ptxA1*/*fim3-1*/*fim2-1* (75.81%). All 62 BP strains were divided into 1, 2, 3, 14, and 52 types at the MGT1, MGT2, MGT3, MGT4, and MGT5 levels, respectively.

**Conclusion:**

This work showed that there may be a link between antimicrobial resistance and alterations in specific molecular types, and the isolates showed a clear change when compared with the vaccine strain and that selection pressure from both antibiotics and immunization may be responsible for driving Chinese BP evolution, and necessitate a reevaluation of the immunization strategy and the development of novel vaccines in China to halt the resurgence and medication resistance of pertussis.

## 1 Introduction

The severity of pertussis, an infectious respiratory disease caused by *Bordetella pertussis* (BP), is high in infants (Payne et al., 2023). Whole-cell pertussis vaccine (WCV) effectively reduced the occurrence of pertussis. In China, WCV was replaced by the less reactogenic acellular pertussis vaccine (ACV), which has been solely used in China since 2013 (Xu et al., 2015). ACV comprises pure BP proteins, especially filamentous hemagglutinin, pertussis toxin promoter (*ptx*) which is the principal pathogenic factor of BP, capable of inducing the immune system to produce antibodies, and pertactin (*prn*) which has robust immunogenicity and is involved in the adhesion mechanism among bacteria, neutrophils, and epithelial cells, with a few additional proteins, including fimbrial proteins (Belcher and Preston, 2015).

Although ACV is effective, the prevalence of BP has been increasing, which is a major global public health concern, especially in industrialized countries (Barkoff and He, 2019). The loss of immunity caused by ACV occurs earlier than that caused by WCV, increasing infection susceptibility in children and adolescents (Belcher and Preston, 2015).

One explanation for the increased circulation of BP is molecular alterations in the pathogen. Various studies have indicated antigenic changes in bacterial virulence genes that may suppress vaccine-mediated immunity against BP (Barkoff and He, 2019). Pertussis vaccination is reported to alter the fimbrial serotype of the circulating strains. Advances in bacterial typing techniques have enabled the elucidation of differential characteristics between vaccination and epidemic strains. In particular, alterations have been detected in the shared virulence genes, such as *ptx* and *prn* (Barkoff and He, 2019). Additionally, genome-based methods, such as multi-locus sequence typing (MLST), multi-locus antigen sequence typing (MAST), and whole-genome sequencing (WGS), have enabled the precise monitoring of BP. Recently, a multi-level genome typing (MGT) method has been developed for BP. This method involves a sequence of MLST methods in which the number of loci is progressively increased (Payne et al., 2023). MGT involves five levels of resolution for BP and can distinguish closely related strains at the smallest scale. After the introduction of acellular pertussis (aP) vaccination, BP strains lacking vaccine antigens, especially prn, have been widely detected (Barkoff and He, 2019). The genotypes of Chinese epidemic BP strains were different from those of vaccine strains (Fu et al., 2023). In the last decade, several countries have reported adaptive alterations in the vaccine-associated genes of circulating BP strains (Saadatian-Elahi et al., 2016). For example, the most common contemporary *ptxA* allele is *ptxA1*, which differs from the *ptxA2* allele commonly observed in the vaccine strain. In several countries, the circulating BP strains have evolved to present a non-vaccine antigen genotype (*ptxA1*/*prn2*/*ptxP3*). Meanwhile, vaccines are not available for the pathogenicity of *prn*-deficient and *fim*-deficient isolates. These isolates exhibit good fitness to vaccine-induced selection pressure (Barkoff et al., 2018; Carriquiriborde et al., 2019). The Chinese pertussis isolates were reported to primarily harbor the *ptxP1* allele (Wang and He, 2015). However, one study demonstrated that mutant strains with the *ptxA1*/*prn2*/*ptxP3* combination were prevalent in China, accounting for 44.8 and 47.7% of isolates in Shenzhen (Zhang et al., 2020) and Shanghai, respectively (Fu et al., 2024).

Erythromycin (ERY), a macrolide antibiotic, is the preferred antibiotic for preventing and treating pertussis. Two mechanisms of ERY resistance have been identified. The first mechanism involves the acquisition of the ERY-resistant methyltransferase gene (*erm*), which confers high levels of resistance (Pechère, 2001). Meanwhile, the second mechanism involves mutations in the 23S rRNA gene, which result in structural alterations, impairing the binding of ERY (Bartkus et al., 2003). Macrolide-resistant BP strains, which were first identified in Shandong Province in 2011, have been widely reported in China (Zhang et al., 2013) with incidence rates of 48.6% (51/105), 72.4% (205/283), and 78.13% (25/32) in Shenzhen (Zhang et al., 2020), Shanghai (Fu et al., 2024), and Beijing (Li et al., 2019), respectively.

Until now, a series of studies about BP strains were reported in China, However, there is inadequate study on the BP pandemic strain in Hebei Province. In order better comprehend the evolutionary traits, and molecular characterization of BP in Hebei, as well as the antimicrobial susceptibility. In this study, 62 BP isolates were collected from Hebei between 2019 and 2020. We systematically analyzed the molecular characteristics, the genomic evolution, and the antimicrobial resistance profiles of those strains.

## 2 Materials and methods

### 2.1 Bacterial isolates

This epidemiological cross-sectional study was performed from 2019 to 2020, and it was approved by the Ethics Committee of the Hebei Province Center for Disease Prevention and Control Review Board (no. 2020–35). Three hundred ninety-seven pertussis positive patients were diagnosed by polymerase chain reaction (PCR) analysis of nasopharyngeal swabs from suspected patients at the surveillance site in Hebei Province. The collection was performed according to the Chinese clinical case diagnostic criteria for pertussis. The samples were then spread onto charcoal agar plates supplemented with 10% defibrinated sheep blood and cephalexin and incubated in a humidified incubator at 35°C for 3–7 days. The presence of BP was verified using a matrix-assisted laser desorption/ionization time-of-flight mass spectrometer (Zybio, China). In total, 62 clinical isolates identified as BP were subcultured on a carbon blood agar plate (OXOID, UK) without cefalexin and stored in a −80°C refrigerator (Thermo, USA) for subsequent use.

### 2.2 Antimicrobial susceptibility testing

Minimum inhibitory concentrations (MICs) of four β-lactam antibiotics [amoxicillin (AMX), meropenem (MEM), ceftriaxone (CRO), and cefotaxime (CTX)], two macrolide antibiotics [ERY and azithromycin (AZM)), one lincosamide antibiotic (clindamycin (CLI)], one quinolone antibiotic [levofloxacin (LEV)], and one sulfonamide antibiotic [sulfamethoxazole (TMZ-SMP)] were determined using the E-test strip (BIO-KONT, China). Culture-positive BP isolates were diluted to match a 0.5 McFarland standard and transferred onto a charcoal agar with 10% sheep blood without cephalexin. Antibiotic-free pills were added to the agar to assess growth and purity. *Streptococcus pneumoniae* (ATCC46916) served as controls. The MICs of antibiotics in the E-test strips were determined using the agar 5 days after inoculation. The Clinical and Laboratory Standards Institute (CLSI) and the European Committee on Antimicrobial Susceptibility Testing (EUCAST) have not established the specific sensitivity and resistance thresholds for BP. Based on pertinent research, an MIC of ≥32 mg/mL was considered as an indication of macrolide resistance. Other antibiotics were evaluated for determining susceptibility by using the breakpoints specified by the 34th edition of the CLSI for *Haemophilus influenzae* (Yang et al., 2015).

### 2.3 DNA extraction and WGS

The genomic DNA of the culture-positive isolates was extracted using the QIAamp DNA mini kit (QIAGEN, Germany), following the manufacturer’s instructions. The purity and concentration of the DNA were assessed using an ultralow volume spectrometer (MIULab, China) and an Invitrogen Qubit 4.0 fluorometer (Thermo, USA), respectively. The DNA samples with qualified concentration and purity were randomly sheared into fragments of approximately 350 bp using ultrasonication. The samples were sequenced using Illumina NovaSeq PE 150.

### 2.4 Single nucleotide polymorphism (SNP) and phylogenetic analyses

The Chinese vaccine strain CS (accession no. GCA_023612135.1) and the international reference strain Tohama I (accession no. GCA_000195715.1) were used as the genome reference sequence. kSNP version 4.1 (Hall and Nisbet, 2023) was used to obtain the alignment of 1,476 SNPs. The number of shared SNPs among 62 BP isolates was 1,269 and was determined based on kmer with an optimum value of 19. The IQ-TREE (Minh et al., 2020) software was used to construct phylogenetic trees based on the maximum-likelihood method with 1,000 ultrafast bootstrap replicates and the model “TN + F + ASC + R3,” which was automatically selected as the best fit. The phylogenetic tree was visualized using TVBOT (Xie et al., 2023).

### 2.5 MLST, MAST, 23S rRNA genes, and Erm genes

MLST of BP was performed by analyzing the following seven specific housekeeping genes using mlst version 2.11 (GiHub, 2022b) to obtain corresponding allele numbers and types: *adk*, *fumC*, *glyA*, *tyrB*, *icd*, *pepA*, and *pgm*. Previously reported different genotype sequences of antigens were retrieved from GenBank and analyzed using HS-BLASTN 2.6.0 (Chen et al., 2015) to type the vaccine antigen genes *ptxP*, *ptxA*, *prn*, *fim2*, and *fim3* and screen 23S rRNA mutations and *erm* presence. The sequences were compared with those curated at the BIGSdb-Pasteur platform (Jolley et al., 2018). CS and Tohama I were included as positive controls.

### 2.6 MGT

The MGT_reads2alleles pipeline (GiHub, 2022a) was applied to extract the alleles from Illumina paired end reads. Genomes that passed the filters were used for further analysis (genome length: 3.5–5 Mb). The generated allele files were uploaded to MGTdb to obtain the MGT assignment. The genome of Tohama I served as the reference genome.

### 2.7 Public genome dataset

This study compared 375 published genomes of Chinese BP strains and global BP isolates. The genomes in the public dataset were sequenced for various purposes and were from 28 countries and eight geographical zones (Appendix Table 1). Unprocessed short-read sequencing data were obtained from the National Center for Biotechnology Information (NCBI) Sequence Read Archive.^1^ The reads were processed using Trimmomatic 0.39 (Bolger et al., 2014) and subjected to *de novo* assembly using Shovill 1.1.0 (GiHub, 2020).

## 3 Results

### 3.1 Laboratory test results and epidemiological characteristics analysis

Sixty-two strains of BP were isolated from nasopharyngeal swabs of 397 children, resulting in a positive percentage of 15.62% (62/397). The vaccination history of the 62 cases was not available. The age range of children with pertussis spans from 1 month to 9 years, exhibiting a male to female ratio of 1:1. Among these, 9 cases (14.52%) are under 3 months, 18 cases (29.03%) are between 3 and 6 months, 9 cases (14.52%) are between 6 and 18 months, 7 cases (11.29%) are between 18 and 36 months, and 19 cases (30.64%) are over 36 months.

### 3.2 Antimicrobial susceptibility and 23S rRNA A2047G mutation analyses

**Figure 1:**
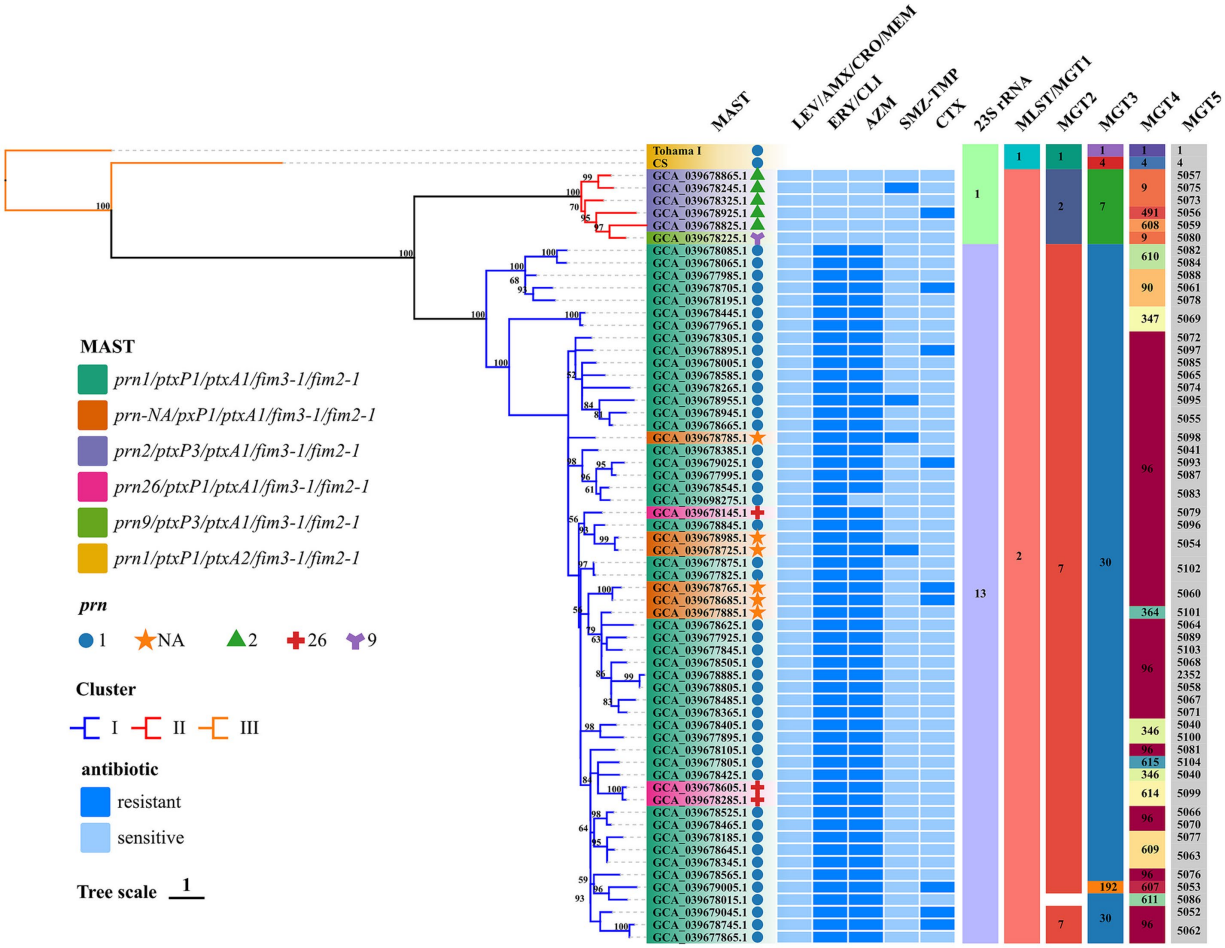
Phylogenomic relationship of 62 Chinese BP isolates. Maximum clade credibility phylogenetic tree for Hebei Province BP isolates in the present studies based on whole-genome SNPs, which included 1,269 SNPs to illustrate the genetic relationship of Hebei Province clinical isolates. Tohama I was used as the outgroup. The ERY resistant lineages I and ERY sensitive lineages II were marked by blue and red, respectively. Bootstrap values of ≥50% were marked at each branch. *Prn* was represented by graphics of different colors and shapes. Various type details of isolates (MLST, MAST, MGT, phenotype of antimicrobial susceptibility and genotype of BP) were shown as color codes per the legends which are on the left side of the figure, Bootstrap values greater than or equal to 50 were shown in numerical form in the figure MGT3 blank space indicates unassigned, the proportion of branch length is expressed as 1.

The E-test revealed 56 ERY and CLI-resistant (MIC >32 mg/mL) isolates, 55 AZM-resistant (MIC >256 mg/mL) isolates, one ERY and CLI-resistant (MIC >32 mg/mL) but AZM-sensitive (MIC = 16 mg/mL) strain (accession no. GCA_039698275.1), 58 TMZ-SMP-sensitive (MIC range = 0.008–1 mg/mL) isolates, and 53 CTX-sensitive (MIC range = 0.064–2 mg/mL) isolates. All isolates were sensitive to AMX, MEM, and CRO (MIC range = 0.064–2 mg/mL). The *erm* gene was not detected in any of the genomes. The A2047G mutation in the 23S rRNA gene was detected in 56 strains tested for sensitivity to ERY (MIC >32 mg/mL) using the E-test. The mutation was not detected in the remaining isolates. Figure 1 shows antimicrobial susceptibility and 23S rRNA mutation analysis results.

### 3.3 MLST, MAST, MGT, and phylogenetic analysis

*In silico* analysis the results of BP were consistent with the BIGSdb-Pasteur results. The distribution of types and analysis results are shown in Figure 1. All test isolates had the *ptxA1*/*fim2-1*/*fim3-1* allele. The *ptxP1* and *ptxP3* alleles were detected in 90.32% (*n* = 56) and 9.68% (*n* = 6) of the isolates, respectively. The following four *prn* allele types were detected: *prn1*, *prn2*, *prn9*, and *prn26*. The predominant *prn* allele was *prn1*, accounting for 79.03% of the *prn* alleles (*n* = 49). Additionally, six isolates exhibited a *prn*-negative phenotype. The following five antigenic genotypes were detected in the isolates according to *prn* and *ptxP* alleles: *prn1*/*ptxP1*/*ptxA1*/*fim3-1*/*fim2-1* (75.81%; *n* = 47), *prn-NA*/*pxP1*/*ptxA1*/*fim3-1*/*fim2-1* (9.68%; *n* = 6), *prn2*/*ptxP3*/*ptxA1*/*fim3-1*/*fim2-1* (8.06%; *n* = 5), *prn26*/*ptxP1*/*ptxA1*/*fim3-1*/*fim2-1* (4.84%; *n* = 3), and *prn9*/*ptxP3*/*ptxA1*/*fim3-1*/*fim2-1* (1.61%, *n* = 1). The genotype of the reference strains was *prn1*/*ptxP1*/*ptxA2*/*fim3-1*/*fim2-1*. All test isolates belonged to MLST sequence type 2 (ST2) and MGT1 ST2. Additionally, two MGT2 STs (MGT2 ST7 and ST2) and three MGT3 STs (MGT3 ST30, ST7, and ST192) were identified. In this study, 13 and 52 types were identified at the MGT4 and MGT5 levels, respectively. One BP (accession no. GCA_039678015.1) was identified as the unassigned type at the MGT3 level. The phylogenetic tree generated using SNPs revealed the following two lineages: Lineage I (56 isolates) and Lineage II (6 isolates). Among the 62 strains, 1,269 shared SNPs were identified. Of these 1,269 SNPs, 373 (29.39%) were core SNPs. The whole-genome sequences of 62 strains were uploaded to the NCBI Sequence Read Archive (BioProject: PRJNA1110622).

### 3.4 Correlation between antimicrobial susceptibility, genotypes, and phylogenetic tree

The antigenic genotypes of all ERY-resistant BP and CLI-resistant BP strains were *prn*1 or *prn*26 or *prn*-NA/*ptxP*1/*ptxA*1/*fim*3-1/*fim*2-1 (88.71, *n* = 55). In addition, the antigenic genotype of the sensitive strains was *prn*9 or *prn*2/*ptxP*3/*ptxA*1/*fim*3-1/*fim*2-1 (11.29%; *n* = 7). The antigenic genotypes of TMZ-SMP-resistant BP were as follows: *prn-NA*/*pxP1*/*ptxA1*/*fim3-1*/*fim2-1* (50%; *n* = 2); *prn1*/*ptxP1*/*ptxA1*/*fim3-1*/*fim2-1* (25%; *n* = 1), and *prn2*/*ptxP3*/*ptxA1*/*fim3-1*/*fim2-1* (25%; *n* = 1). ERY-resistant BP belonged to Lineage I, while ERY-sensitive BP belonged to Lineage II. Comparative analysis of several types revealed that 62 BP isolates were consistent and belonged to MLST ST2 and MGT1 ST2. *ptxP*1 was mostly associated with MGT2 ST7 in the 56 strains with 23S rRNA allele 13 harboring the A2047G mutation. Furthermore, this mutation did not occur in six strains with 23S rRNA that had allele 1, and *ptxP*3 was mostly linked to MGT2 ST2. *ptxP1*-ERY-resistant BP strains belonged to MGT3 ST30 (98.21%; *n* = 55), while *ptxP3*-ERY-sensitive BP strains belonged to Lineage II and MGT3 ST7. At the MGT4 level, all *ptxP3*-ERY-sensitive BP strains belonged to MGT4 ST9 (66.67%; *n* = 4), MGT4 ST491 (16.67%; *n* = 1), and MGT4 ST608 (16.67%; *n* = 1). These strains can be separated from *ptxP1*-ERY-resistant BP strains. Although *prn*-negative strains could be detected at the MGT4 and MGT5 levels, they could not be distinguished at the MGT1, MGT2, and MGT3 levels and hence belonged to MGT1 ST2, MGT2 ST7, and MGT3 ST30. The STs at the MGT4 level were MGT4 ST96 (83.33%; *n* = 5) and MGT4 ST364 (16.67%; *n* = 1). Meanwhile, the STs at the MGT5 level were MGT5 ST5054 (33.33%; *n* = 2), MGT5 ST5060 (33.33%; *n* = 2), MGT5 ST5098 (16.67%; *n* = 1), and MGT5 ST5101 (16.67%; *n* = 1). At the MGT5 level, the STs of four strains of TMZ-SMP-resistant BP were ST5095, ST5054, ST5075, and ST5098. The STs of nine CTX-resistant strains were ST5052, ST5053, ST5056, ST5061, ST5062, ST5093, and ST5097.

### 3.5 Phylogenetic tree for the correlation between Hebei and global BP isolates

**Figure 2:**
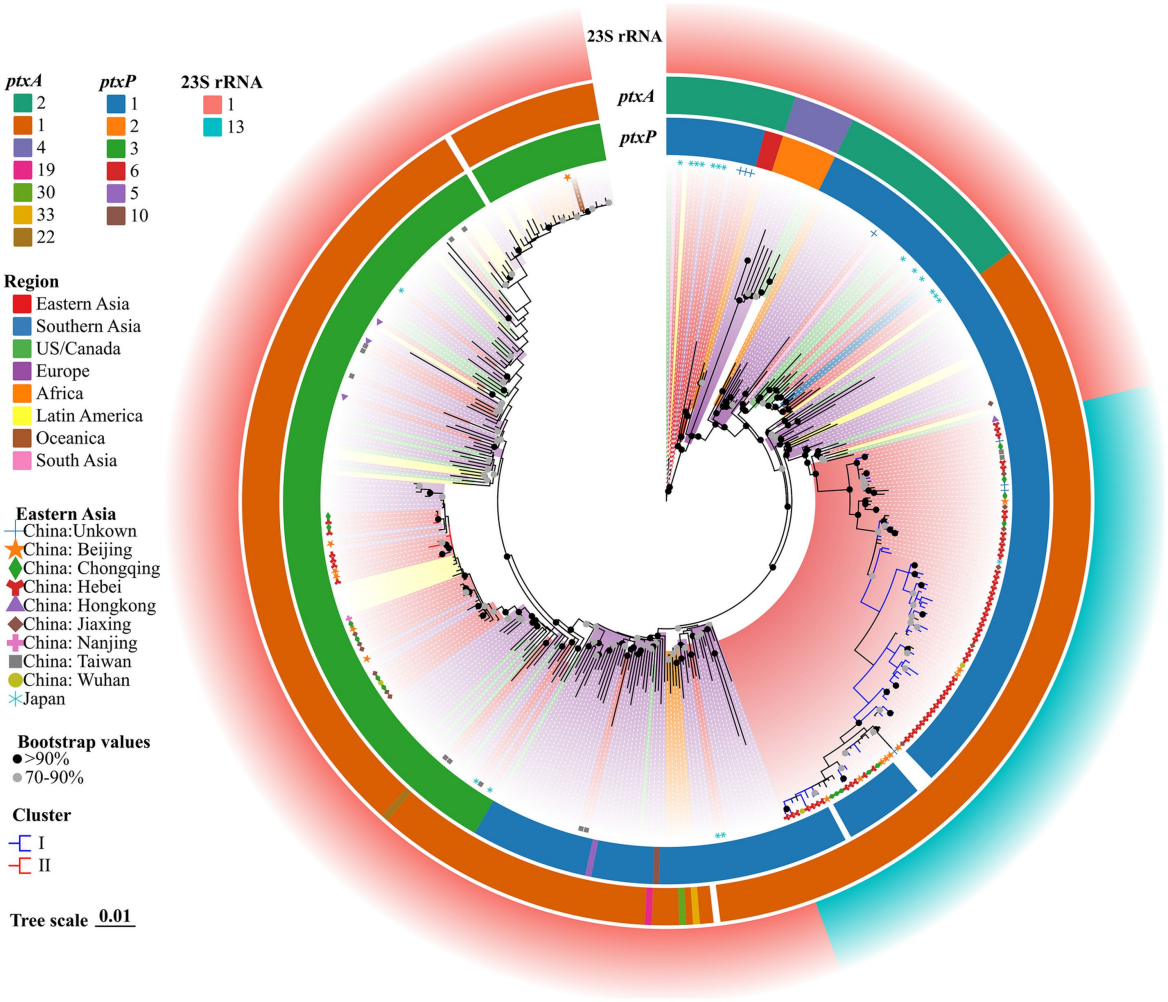
ML phylogenetic tree of 62 Hebei, China, and 313 global BP strains, 2019–2020. Tohama I was used as the outgroup. The ERY resistant lineages I and ERY sensitive lineages II in this study were marked by blue and red, respectively. The BP of eight regions were represented in different colors. Regions in Eastern Asia were represented by graphics of different shapes and colors. Isolate details (*ptxA*, *ptxP* and 23S rRNA allele type) were shown as color codes as per the legends. The blank space in the figure represents the gene deletion corresponding to the BP. Bootstrap values greater than or equal to 70 were shown in the figure as circles of two different colors, the proportion of branch length was expressed as 0.01.

The genomes of all 375 strains (62 BP strains from this study and 313 strains from the public dataset of global isolates) were matched using the genome of Tohama I as the outgroup with KSNP4. In total, 10,494 SNPs, including 3,897 core SNPs, were identified. Additionally, 56 strains of *ptxP1*-ERY-resistant BP from Hebei were closely related to other isolates from Eastern Asia, including China, several cities, and one Japan BP (GenBank accession no. GCA_019974435.1). However, these 56 strains differed from other international strains isolated from the United States, Europe, Australia, Africa, Iran, Israel, Argentina, Brazil, and other regions and countries (Figure 2). Six strains of *ptxP3*-ERY-sensitive BP from Hebei were closely linked to other isolates from Europe, Latin America, and China (Beijing and Chongqing). Among 375 BP strains, the strains with the 23S rRNA allele A2047G mutation were evolutionary branches from Eastern Asia that were closely connected to 56 strains of *ptxP1*-ERY-resistant BP. Compared with the BP reference strain Tohama I harboring *ptxP1* and *ptxA2*, the Hebei isolates exhibited a diverse evolution of *ptxP* and *ptxA* alleles, while the *prn* genes underwent varying degrees of deletion.

## 4 Discussion

Active monitoring of pertussis has revealed that the incidence rate of pertussis is increasing in Hebei Province (Zheng et al., 2024). Therefore, there is a need to determine the cause of increased prevalence rates of pertussis. Previous studies have demonstrated that the resurgence of pertussis can be attributed to BP isolates harboring antigen gene mutations and their distinct characteristic relative to the vaccine strain (Miyaji et al., 2013). To understand the potential causes of pertussis recurrence in Hebei, this study investigated the genetic diversity and molecular epidemiological characteristics of 62 BP strains collected between 2019 and 2020.

ERY-based macrolide antibiotics are the first treatment choice for pertussis (Zhang et al., 2022). In this study, antibiotic sensitivity analysis of 62 strains of BP isolates from Hebei Province revealed that Hebei isolates exhibited high resistance to macrolides, Allele 13 of the 23S rRNA locus which harbored the A2047G mutation was unique (Bridel et al., 2022) for ERY-resistant BP. Additionally, *ptxP1*-ERY-sensitive BP strains harbored the 23S rRNA A2047G mutation, and *ptxP3*-ERY-sensitive BP strains did not harbor the A2047G mutation. The incidence rates of ERY and AZM resistance were more than 90%. This indicates that *ptxP1*-ERY-sensitive BP strains harboring A2047G mutation were widely distributed in Hebei. Hence, the high incidence rate of macrolide resistance among BP strains in China must be addressed. The incidence rate of CLI resistance was 90.32%. CLI belongs to the lincosamide class of antibiotics with similar mechanisms as macrolides. Thus, the incidence of CLI resistance was like that of ERY resistance. One isolation in this study (GCA_039698275.1) was resistant to ERY and CLI (MIC >32 mg/mL) but sensitive to AZM (MIC = 16 mg/mL). Previous studies have reported two strains that were resistant to ERY and AZM but sensitive to clarithromycin (Zhang et al., 2022). The 23S rRNA A2047G mutation was assumed to be the primary etiological factor for the resistance of BP to macrolides (Barkoff and He, 2019). However, the precise mechanism underlying the variations in ERY and AZM resistance has not been elucidated. SMZ-TMP was traditionally considered the secondary therapeutic option (Zhang et al., 2020). In this study, the incidence rate of SMZ-TMP sensitivity was 93.55%. In patients with pertussis exhibiting resistance to macrolides, SMZ-TMP must be recommended for the treatment of pertussis caused by macrolide-resistant strains in children aged over 2 months in China (Group et al., 2024). AMX, MEM, CRO, and CTX belong to β-lactam antibiotics. All 62 BP isolates were sensitive to AMX, MEM, and CRO. However, the incidence rate of CTX sensitivity was 85.48%. This suggested that these four drugs can be used to treat patients with pertussis. Early studies did not support the use of β-lactam antibiotics to treat pertussis. However, some studies suggested the effectiveness of β-lactam antibiotics in pertussis. The *in vitro* sensitivity to different β-lactam drugs may significantly vary (Yao et al., 2024). In this study, 62 BP strains were sensitive to the quinolone drug LEV. However, LEV exerts toxic effects in children and can be used to treat only teenagers or adults (Yao et al., 2024). Further studies are needed to assess the viability of β-lactam antibiotic treatment for the clinical management of patients, especially infants and young children, infected with macrolide-resistant strains. Furthermore, the effectiveness of quinolone antibiotics must be evaluated through clinical trials.

Globally, *ptxP3* strains are commonly detected. In contrast, this study revealed that *ptxP1*-Macrolide-resistant BP strains were common in Hebei with 90.32% of the isolates harboring the *ptxP1* allele (Barkoff and He, 2019; Xu et al., 2019). The prevalence of *ptxP1*-Macrolide-resistant BP strains in China may be attributed to antibiotic selection. The *ptxP1*-Macrolide-resistant BP strains have several advantages over *ptxP3*-Macrolide-sensitive BP strains in the population in which the usage of antibiotics is high. The MAST analysis categorized 62 strains of BP into six distinct categories with most strains (75.81%) exhibiting the antigen genotype *prn1*/*ptxP1*/*ptxA1*/*fim3-1*/*fim2-1*. Both the Chinese vaccination strain CS and the international strain Tohama I harbored the *prn1*/*ptxP1*/*ptxA2*/*fim3-1*/*fim2-1* genetic markers. All BP samples in this study contained the *ptxA1*/*fim3-1*/*fim2-1* genotype. However, in this study, all BP isolates carried the *ptxA1/fim3-1/fim2-1* alleles, while both *prn* and *ptxP* exhibited distinct variations in their alleles. Furthermore, the *prn* gene was demonstrated to exhibit a range of allelic variations in this study. The polymorphism of *prn* gene and the transmission of *prn*-defective isolates may represent the selective avoidance of vaccine-induced immunity and antibiotics by bacteria (Xu et al., 2019). *prn* deficiency has been documented in several areas when ACV containing *prn* was used. In particular, the incidence of *prn*-deficient strains was high in Australia and some other nations (Barkoff and He, 2019). Six *prn-*deficient strains were identified in this research and there had studies discovered that *prn*-negative strains exhibit increased adaptation in mouse models (Safarchi et al., 2015). Studies have shown that *prn-*deficient strain was the most suitable genotype in the era of aP vaccine and after the introduction of aP vaccine, the average fitness of *prn-*deficient strain was 1.26 times than those strains which carrying *prn* gene (Lefrancq et al., 2022), so it is expected that the frequency of *prn-*deficient strains will further increase in the future.

The MLST and MAST of Hebei isolates differed from those of vaccination strains. However, the resolution was low. To differentiate between closely related isolates in the BP population, the genome must be widely covered due to its limited genetic diversity. SNP-based evolutionary trees can provide an accurate representation of phylogenetic evolution and linkages. This study revealed three lineages, which included the reference strain. Furthermore, the strains found in Hebei Province shared a distant genetic link with the reference strain, indicating that the epidemic strain has undergone varied degrees of mutation when compared with the vaccination strain. Compared with the international epidemic BP strain, 56 strains of *ptxP1*-ERY-resistant BP formed a distinct branch in the Eastern Asia region where China is located. Meanwhile, six strains of *ptxP3*-ERY-sensitive BP were closely related to individual strains in parts of Europe and economically developed cities in China. MGT supports multi-level resolution. The MGT5 level (BP core gene MLST) has the same resolution as an existing entire gene. MLST scheme maintains the standardization that is inherent in a cgMLST scheme (Payne et al., 2023). MGT1 and MGT2 were compatible with the MLST and BPagST results, respectively. The resolution of BP genotyping was significantly high at the MGT4 and MGT5 levels, which can be used to identify outbreaks and track their origins.

In conclusion, the epidemic trend of antimicrobial susceptibility of BP in Hebei Province is consistent with that of BP in China in recent years (Li et al., 2019; Xu et al., 2019; Zhang et al., 2022; Fu et al., 2024). The antigen genotype of circulating BP stains in Hebei Province is consistent with the epidemic trend of BP in Midwest China in recent years (Xu et al., 2019; Zhang et al., 2022) but varied from the epidemic trend of BP in the southern part of the relatively developed regions, such as Shenzhen (Zhang et al., 2020) and Shanghai (Fu et al., 2024), and industrialized nations, including Australia and the United States (Barkoff and He, 2019). Generally, *ptxP3*-BP is more common in countries that have used ACV than *ptxP1*-BP, which is common in countries that have used WCV. WCV was replaced in China in 2012 and has been the sole vaccine used in China since 2013. The implementation of WCV in China was delayed by a decade when compared with that in industrialized countries, such as the United States and Australia. WCV is implemented in developed areas of China, such as Shanghai but is less commonly implemented in underdeveloped areas. Thus, *ptxP3* strains may not have had enough time to replace *ptxP1* strains in China (Xu et al., 2019). This can explain the differential circulating strains between some Chinese cities. Compared with those of BP vaccination strains, important virulence protein genes and core genomes of Hebei isolates have significantly evolved. The findings of this study reveal potential gaps in BP genetic diversity in Hebei Province and provide essential reference values for improving the pertussis vaccine in China. Nonetheless, the current findings may be constrained by the limited number of strains examined, and subsequent research should encompass a broader range and greater volume of sampling to validate the conclusions. Furthermore, thorough screening for potential mechanisms of antibiotic resistance commonly associated with individuals suffering from pertussis is necessary, and thereby elucidate the relationship between the development of antimicrobial resistance in BP and alterations in genotypes.

## Data Availability

The datasets presented in this study can be found in online repositories. The names of the repository/repositories and accession number(s) can be found in the article/Supplementary material.

## Footnotes

^1^ https://www.ncbi.nlm.nih.gov/sra
